# 外加场分离技术与微流控技术联用在微纳尺度物质分离中的研究进展

**DOI:** 10.3724/SP.J.1123.2020.12032

**Published:** 2021-11-08

**Authors:** Jiaxuan CUI, Lu LIU, Donghao LI, Xiangfan PIAO

**Affiliations:** 1.延边大学理学院化学系, 吉林 延吉 133002; 1. Department of Chemistry, School of Science, Yanbian University, Yanji 133002, China; 2.延边大学工学院电子系, 吉林 延吉 133002; 2. Department of Electronics, School of Engineering, Yanbian University, Yanji 133002, China

**Keywords:** 微流控, 微纳尺度物质, 主动场分离, 综述, microfluidic, micro/nanoscales, active field separation, review

## Abstract

微纳尺度物质的分离和分选在精准医学、材料科学和单细胞分析等研究中至关重要。精准、高效和快速的分离微纳尺度物质能够为癌症的早期诊断、生物样品检测和细胞筛选提供重要帮助,其中基于外加场分离技术的分离微纳尺度物质因可以对微纳尺度物质高效在线分离和分选,被广泛应用于微纳米颗粒、外泌体以及生物细胞的分离工作中,而目前多数外加场分离技术存在装备繁琐和样品消耗大等问题。微流控技术是一种通过制作微通道和微流控芯片操纵微小流体对微纳尺度样品组分进行分离的技术,因具有快速检测、高通量、在线分离、集成性高、成本低等优势现被应用于微纳尺度物质分离分析中,是一种微纳尺度物质分离的有效方法,通过在微流控芯片上设计不同的通道及外部配件提高主动场对微纳尺度物质分离效率。外加场分离技术与微流控技术联用可以实现微纳尺度物质的无损、高效、在线分离。该综述主要概述了近年来在微流控芯片上依托流动场、电场、磁场及声场等外加场分离技术来提高对微纳尺度物质分离效率的研究现状,并将各个外力场对单细胞、微颗粒等微纳尺度物质的分离进行分类介绍,总结各自的优缺点及发展应用,最后展望了外加场分离技术与微流控技术联用在应用于癌细胞的早期筛查、精确分离微尺度物质领域的未来发展前景,并提出联用技术的优势和未来应用等。

## Abstract (en)

The micro/nanoscales concerns interactions of entities with sizes in the range of 0.1-100 μm, such as biological cells, proteins, and particles. The separation of micro/nanoscales has been of immense significance for drug development, early-stage cancer detection, and customized precision therapy. For example, in recent years, rapid advances in the field of cell therapy have necessitated the development of simple and effective cell separation techniques. The isolation technique allows the collection of the required stem cells from complex samples. With the development of materials science and precision medicine, the separation of particles is also critical. The key physicochemical properties of micro/nanoscales are highly dependent on their specific size, shape, functional group, and mobility (based on the charged characteristics), which control their performance in the separation system. The current demand has made the simultaneous innovation of a separation system and an on-line detection platform imperative. Accordingly, various analytical methods involving the use of external forces, such as the flow field, magnetic field, electric field, and acoustic field, have been used for micro/nanoscales separation. Based on the physical and chemical parameters of the separation materials, these analytical methods can select different external force fields for micro/nanoscales separation, enabling real-time, accurate, efficient, and selective separation. However, at present, most of the applied field separation technologies require complex equipment and a large sample amount. This makes it crucial to miniaturize and integrate separation technologies for low-cost, rapid, and accurate micro/nanoscales separation. Microfluidic technology is a representative micro/nanoscales separation technology. It requires only a small volume of liquid, making it cost-effective; its high throughput enables continuous separation and analysis; its fast response in a microchip can allow many reactions; and finally, the miniaturization of the device allows the coupling of multiple detectors with the microchip. With the continuous growth and progress of microfluidic technology, some microfluidic platforms are now able to achieve the non-destructive separation of cells. They also enable on-line detection, offer high separation efficiency, and allow rapid separation for different biological samples. This review primarily summarizes recent advances in microfluidic chips based on flow field, electric field, magnetic field, acoustic field, and field separation technologies to improve the micro/nanoscales separation efficiency. This review also discusses the various external force fields of micro/nanoscales, such as a microparticle, single cell separation of substances classified introduction, and summarizes the advantages and disadvantages of their application and development. Finally, the prospect of the combined application of external field separation technology and microfluidic technology in the early screening of cancer cells and for precise micro/nanoscales separation is discussed, and the advantages and potential applications of the combined technology are proposed.

微纳尺度物质(尺寸分布为0.1~100 μm的细胞、生物大分子、合成颗粒物、胶体等)的分离分选在生命科学、材料科学和环境科学等领域中至关重要,分离微纳尺度物质将在靶标筛查、个体化差异、新药开发、个体化精准治疗等方面具有巨大意义^[1,2,3]^。目前,主要根据微纳尺度物质表面的物理化学性质如尺寸、形状、电荷、质量等不同对其进行分离分选^[4]^。现有的分离微纳尺度物质方法可以分为两大主流。一种是基于目标物尺寸差异在通道和流体的共同作用下的路径不同,从而实现分离的被动式分离技术,如确定性侧向位移、惯性聚焦、超滤法、离心法等。虽然被动式分离技术取得了一定进展,但其普遍存在分离度低、易堵塞通道、难以实现在线分离检测的问题^[5,6,7]^。第二种分离方法是根据混合目标物中不同目标物所具有的物理化学性质不同,通过添加不同的外力场使目标物在分离系统内部的运动行为发生改变,从而实现分离的主动式分离技术,外加力场类型有电场^[8]^、磁场^[9]^、流场^[10]^及声场^[11]^等,这些主动式分离技术可以实现对微纳尺度物质的在线分离和分选,并且在微颗粒、外泌体、病毒及单细胞分离工作中展现出重要的作用^[8,12-15]^。然而如何使这些分离技术小型化、集成化、易操作仍然是现今待解决的问题。

微流控技术(microfluidics)也被称为芯片实验室(lab chip),起源于1990年Manz等^[16]^提出的“微全分析系统”(miniaturized total chemical analysis systems, μTAS),指的是通过制作微管道(尺寸为数十到数百微米)或微流控芯片来操纵微小流体(体积为pL~μL)并对微尺度物质样品组分进行分离的技术,是一种主要针对微纳尺度物质分离的有效方法。微流控技术实现了对微纳尺度物质的精准、高通量、在线分离,可以用最少的试剂、时间和成本完成分离任务且具有微型化、集成化、成本低廉、高通量等特征^[17,18]^。随着微流控技术的发展,利用微纳尺度物质的不同性质,制作特殊结构的微流控芯片装置,以提高对微纳尺度物质的分离效率,更具有针对性,微流控芯片的生物相容性提高了其在生物细胞操作^[19,20]^和分析^[21,22]^中的应用,同时在微流控技术方面可以使复杂分析方案合理化,显著减少样品体积和试剂成本,在处理微量样品时具有降低成本、降低危害、提高分辨率等优势。随着微流控技术对微纳尺度物质分离发展的不断增长与进步,针对细胞、颗粒物等微纳尺度物质的分离在医疗领域^[23,24,25]^、生物化学领域^[24,26-28]^等起到了至关重要的作用。利用这些优势可以将基于外加场的分离技术与微流控技术进行联用,制备所需的微流控芯片^[29]^,针对不同特性的样品施加外部力场,比如电场^[30,31]^、磁场^[32,33]^及声场^[11]^等来对混合样品组分进行精准分离。

本文主要概述了在微流控芯片上依托流动场、电场、磁场及声场的主动分离技术来提高分离效率的研究现状,并探讨对生物细胞的富集与混合颗粒物的有效精准分离的发展与应用。

## 1 流场场流分离技术

流场场流分离技术(flow field-flow fractionation, FIFFF)是各种场流分离技术中使用最通用的一种技术,其中非对称流场流分离技术(asymmetrical flow field-flow fractionation, AF4)是1987年由Wahlund和Giddings提出的一种流场分馏技术^[34]^,目前被广泛应用。该技术将非特异性的相互作用减少到最低限度,并具有分辨率高的优点^[35,36]^,在FIFFF通道内,外加力场为垂直于流道方向的横向流,样品在横向流的驱动下与自身扩散力之间达到一个平衡,各组分在通道内壁上产生分布差异,其中,小尺寸样品在积聚壁上形成的分布层要高于大尺寸颗粒,这时流动场在通道内流动时,分布层较高的小尺寸颗粒要比大尺寸颗粒更早的洗脱,从而实现分离^[37]^。FIFFF没有固定相,对样品施加的剪切力和机械应力较小,使它成为一种温和的分离技术,现已广泛应用于分离和表征不同尺寸和不同形状的颗粒、细胞、蛋白质或DNA等物质^[38,39]^。

Dou等^[40]^利用非对称流场流分离技术在线耦合紫外(ultraviolet absorption detecto, UV)、多角度光散射(multiangle light scattering, MALS)和荧光(fluorescence, FS)探测器对蛋黄血浆进行分离和表征。利用蛋黄血浆作为AF4的载体液,评价了AF4对蛋黄血浆中的可溶性蛋白、低密度脂蛋白(low density lipoproteins, LDL)及其聚集物进行高效快速分离和表征的实用性。同时研究了低密度脂蛋白在卵黄血浆中的聚集行为,利用程序交叉流的AF4具有提高检测能力、降低样品消耗和减少分析时间等优点。结果证明,AF4适用于尺寸分布范围较大目标物的分离和表征,如蛋黄血浆。该团队还利用AF4结合多角度光散射和差分折射探测器(differential refractive index, DRI)对淀粉的分离和表征进行了深入研究^[41]^,为今后更好地研究淀粉结构-功能的关系提供重要信息。

Ashby等^[42]^利用流场场流分离技术结合离心分离技术,建立了一种基于相对解离率的冠状蛋白鉴定方法,用来筛选纳米颗粒和蛋白质之间的相互作用。该方法将超顺磁氧化铁纳米颗粒(superparamagnetic iron oxide nanoparticles, SPION)和免疫球蛋白G(lgG)在人血清中进行孵育,再利用F4和离心法分离出与SPION亲和力较好的蛋白质,F4以较快的速度洗去与纳米颗粒相互作用的蛋白质,解决了当纳米粒子进入到生物基质时,基质表面形成的蛋白冠对纳米粒子在生物系统中的后续行为影响,有助于研究蛋白质冠的时间分布及其在生物基质中的演化,以及高通量分析蛋白质冠与粒子特性相关的动态特征。

Adkins等^[43]^将纳米颗粒跟踪技术(nanoparticle tracking analysis, NTA)与AF4耦合得到AF4-NTA技术,弥补了NTA在线检测器存在检测范围窄、流量小和压力阈值低等问题。AF4-NTA作为一项对混合物中不同粒子数的纳米材料进行高效精确粒子计数的技术,利用合理的分流设计,对尺寸为50、100和200 nm的聚苯乙烯混合物进行分离分析,同时在线对混合物中不同纳米尺寸目标物进行精确地颗粒计数。

目前,AF4在不断地进步与发展,无论在化学分离领域或者生命科学等其他重要领域都显示出了巨大的潜力,它可以利用温和分离且装置结构简单等特性,与不同的检测器进行耦合,为生物治疗和纳米颗粒分离表征提供技术支持。在未来,AF4温和分离特性优势与微流控的通道小型化、节约试剂和节约样品成本的优势相结合,成为一种高度灵活和具备高分辨率的分离技术,具有巨大的发展前景。

## 2 基于外加电场的分离技术

近年来,越来越多的科学家利用电场对微纳尺度物质进行分离分析。主要分离原理是根据目标物尺寸、大小和带电荷量等特性的不同,通过调节电参数使其在分离系统内的运动行为发生变化,达到对生物细胞和颗粒物等微纳尺度物质的操纵与分离。常见的外加电场的分离方法分为4种(见图1),分别为毛细管电泳^[44]^、介电泳(dielectrophoresis, DEP)^[29,31]^、电场场流分离^[45,46,47]^、电渗驱动^[48]^。

**图1:**
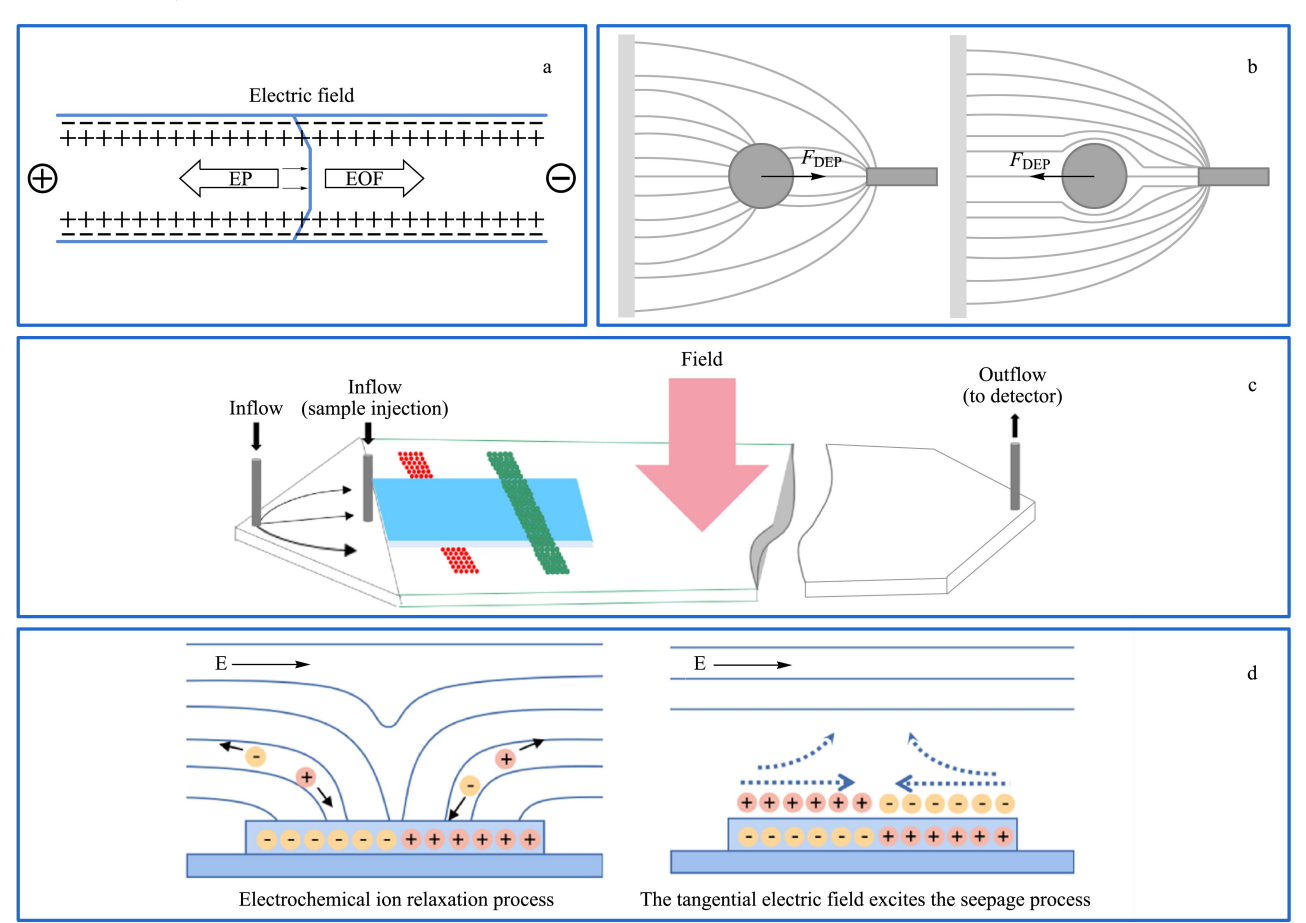
目前被广泛使用的4种电场分离技术

### 2.1 毛细管电泳

根据在一定的电场作用下带电粒子在介质中定向迁移的性质,利用毛细管电泳技术在两端施加高压电场对微纳尺度物质的分离分析满足当今高效快速的分离需求^[49,50,51]^。毛细管内壁与缓冲溶液的界面上形成双电层,在高压电场的驱动下形成定向运动的电渗流。如图1a所示,带电粒子根据自身的电泳力和电渗流力差异实现分离^[52]^。毛细管电泳具有分析快速灵敏、样品消耗量少、分离效率高等优点,在药物分析、环境监测、食品检测中应用广泛^[50,52-55]^。将毛细管电泳技术与微流控技术联用,即微流控芯片电泳(microchip electrophoresis, MCE)是近年被广泛应用的一种新型分离技术,具有低成本、分辨率高、快速等优点^[56,57]^,被广泛用于微纳尺度物质的分离分析中。Zhang等^[58]^利用微流控芯片电泳技术对大肠杆菌、金黄色葡萄球菌和鼠伤寒沙门氏菌3种细菌进行定量检测,有助于对人工污染的生食肉类中的致病菌进行分析,结果显示MCE技术具有灵敏度高、速度快、试剂消耗少和操作迅速等优点,是一种有效、可靠的食品安全评价方法。

Jeon等^[59]^开发了一种基于压力驱动流诱导电泳的连续分离方法,如图2a所示,在微流控装置内,混合的微纳尺度物质受到来自流体的驱动力、电渗流带来的阻力和电泳力为主导的3种合力,根据其自身受电场影响下的电泳迁移率不同而进行有效分离,分离效率可达97%。

**图2:**
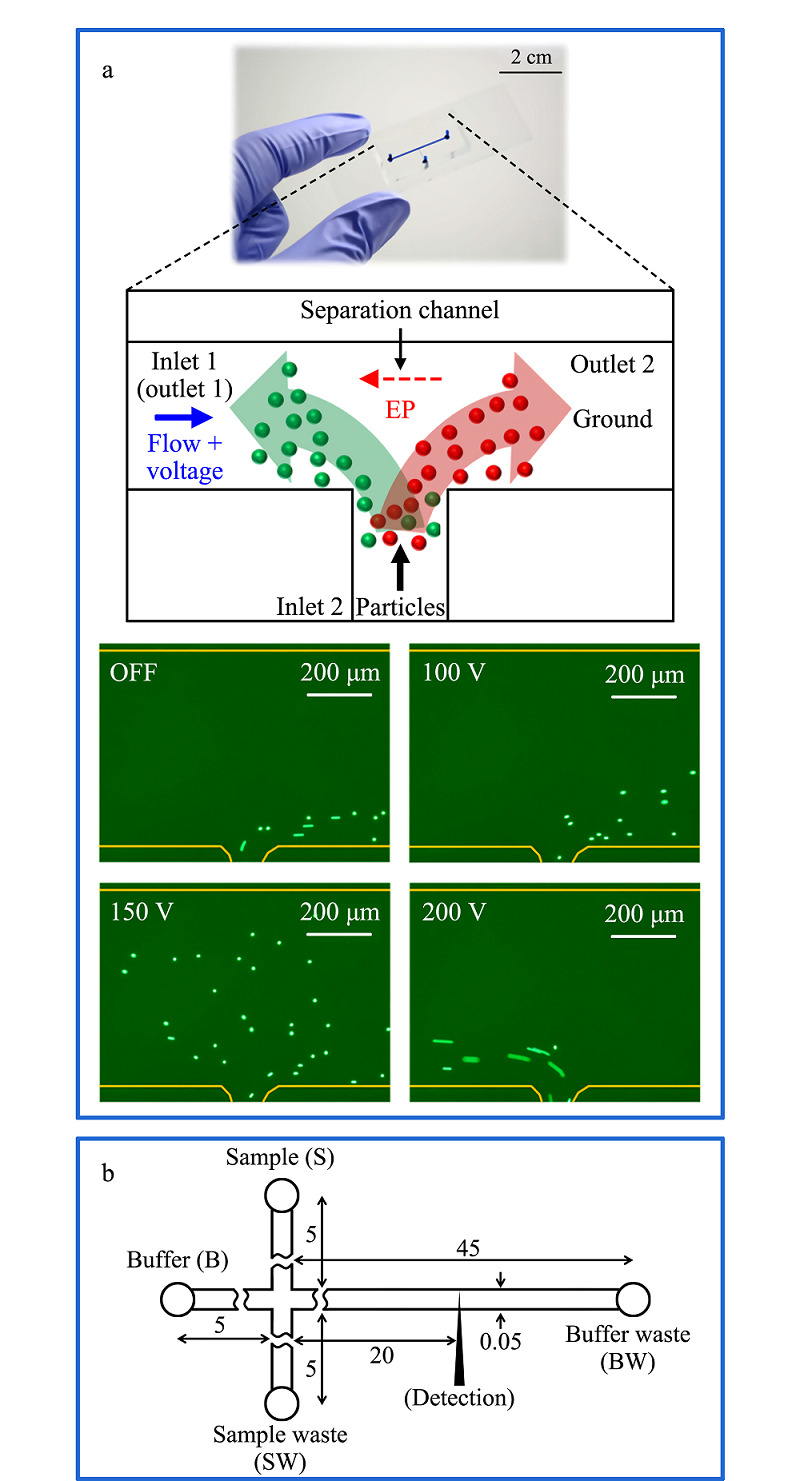
基于微流控芯片电泳的分离系统

蔡绮丹等^[60]^为了验证阿霉素这一常用的蒽环类抗肿瘤药物是否与谷胱甘肽存在结合,利用微流控芯片电泳技术微型化集成化等优点。如图2b所示,利用简化的Hummel-Dreyer芯片毛细管电泳法,考察了阿霉素与还原性、氧化型谷胱甘肽的亲和作用,最终得到了谷胱甘肽自身与阿霉素无亲和作用这一结论,为抗肿瘤药物的研发提供了理论支持。综上所述,毛细管电泳技术与微流控技术的联用在医疗、食品和生物等各个领域都有很大的发展前景。

毛细管电泳技术与微流控技术的联用同时具备毛细管电泳技术无标记和对细胞等无损伤的优势,联用微流控技术的高效、微型化精准分离,解决了传统毛细管电泳技术装置繁琐等问题,具有广阔的应用前景。

### 2.2 介电泳

介电泳由Pohl^[61]^在20世纪50年代首次研究提出,指可极化粒子在非匀强电场中将会受到极化作用进而产生偶极矩,偶极矩与非匀强电场之间产生介电泳力。粒子在该体系内将会受到指向场最大的力正介电泳力(positive dielectrophoresis, pDEP),或者远离场的最大力负介电泳力(negative dielectrophoresis, nDEP),如图1b所示。介电泳操纵粒子具有集成化、操作方便、成本低廉等优势,已广泛用于分离微颗粒和细胞^[62,63,64,65]^。但是介电泳如果在强电场条件下对生物样品进行分离,则会导致生物样品在电场内受焦耳热的影响直接死亡或产生不可逆的损伤^[65]^。因此,利用微流控装置产热少、高通量和成本低等优势,将介电泳技术与微流控技术联用,可以实现对生物样品无损和高效的分离^[31]^。

为了解决多组分样品的同时富集,Zhao等^[66]^研制了一种新型微流控装置,在直流介电泳(direct current-DEP, DC-DEP)提供的非匀强电场条件下,调节外加电场的电参数和流动相悬浮液的电导率,实现对大小相近但介电特性不同的微纳米混合颗粒物的分离。

Zhao等^[67]^还制作了新型交流介电泳(alternating current-DEP, AC-DEP)微流控芯片,芯片同时将两个电极嵌在相对侧壁上的一组不对称孔内,使产生不均匀的电场。如图3a所示,生物细胞等样品通过微流控装置内时,利用液聚焦使样品在同一水平线移动,聚焦后的样品进入到DEP电场范围内时,样品受pDEP和nDEP的影响分别向两侧孔内移动。该实验研究了活酵母细胞和死酵母细胞在不同离子浓度、电导率、交流电场频率下的DEP行为。与直流介电泳不同的是,该装置利用调节交流电频率、电压等参数,成功分离大小相近但介电常数不同的活酵母细胞和死酵母细胞。该微流控装置制作简单,设计可以避免焦耳热效应,且能诱导非均匀电场产生强梯度,可用于分离尺寸相近的纳米颗粒。

**图3:**
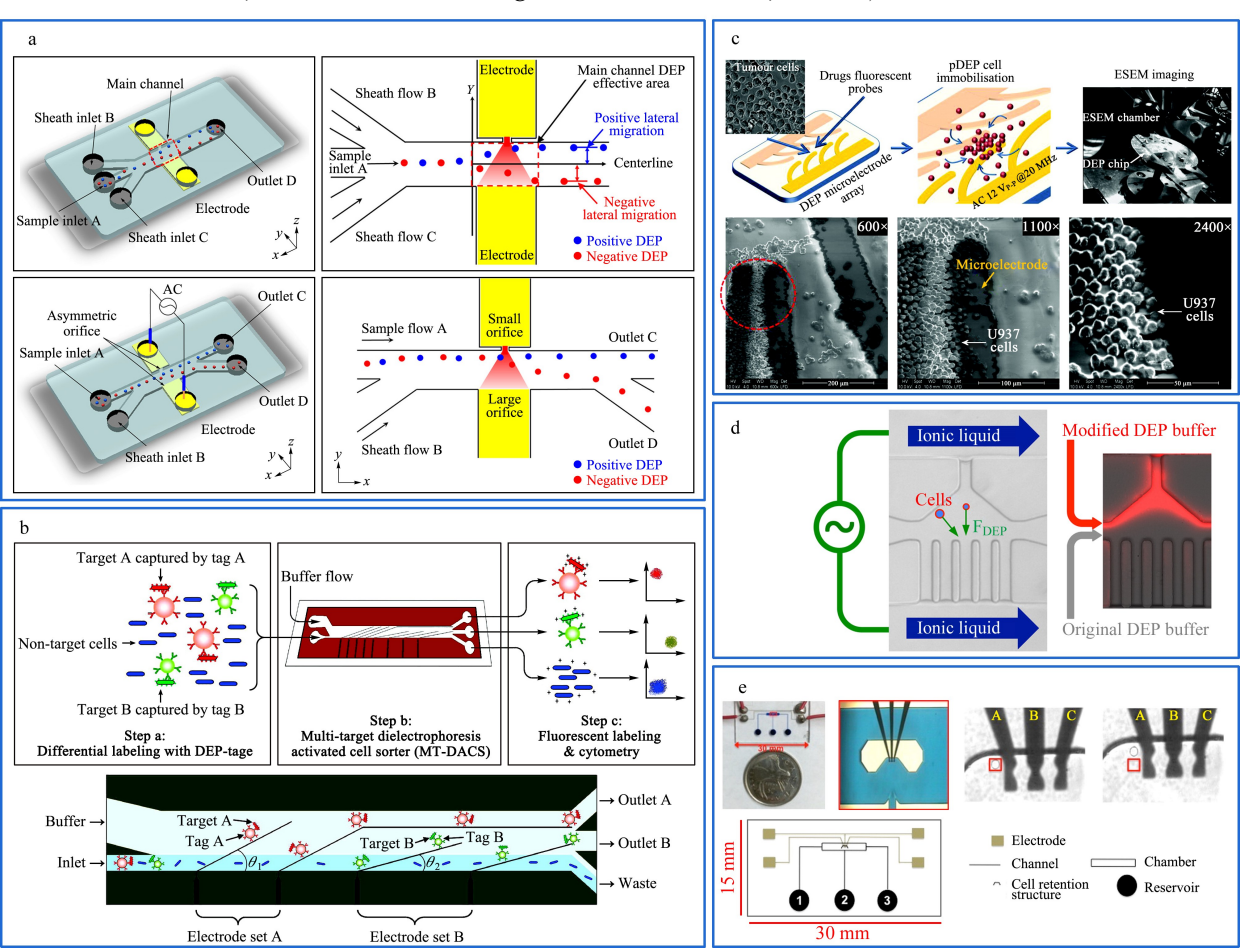
基于介电泳技术的微流控分离系统

Kim等^[68]^使用独特的合成介电泳标记来明确多种细胞类型,在微流控装置的内部通道结构上放置两个具有不同角度的倾斜电极,利用倾斜电极提供非均匀电场,如图3b所示。在非匀强电场下,混合样品中不同尺寸的颗粒物在装置内产生的运动行为不同,实现了高分辨率和高吞吐量的分离效果。

Khoshmanesh等^[69]^设计了一种非黏附的脂质捕获DEP系统,如图3c所示,利用光刻技术在玻璃基板上制备了DEP微电极阵列,避免了生物细胞的污染。基于芯片的阵列应用于捕获人白血病细胞的环境扫描电子显微镜(environmental scanning electron microscope, ESEM)分析。这项工作验证了DEP细胞保留和捕获技术。利用DEP芯片对人白血病细胞进行捕获,同时在ESEM上对单个非贴壁细胞进行高分辨率分析,达到了造血肿瘤和干细胞的水动力捕获和长期动态分析。

Sun等^[70]^开发了一个具有自组装液体电极的新型DEP微流控装置,如图3d所示。利用室温离子液体形成的液体电极与DEP缓冲溶液耦合,再利用外部电场所施加的电压,提高微流控芯片内的电导率,产生电场梯度以对芯片内的细胞与粒子进行高效分离,利用自组装的液体电极DEP微流控装置成功分离聚苯乙烯珠与PC-3细胞、存活与凋亡的PC-3细胞以及人脂肪干细胞(adipose-derived stem cells, ADSCs)与MDA-MB-231癌细胞。该装置具有成本低、分离效率高等优点,在细胞分离实验中具有巨大潜力。

Khamenehfar等^[71]^利用介电泳对液体介质中悬浮的可极化粒子具有可操纵性的特点,制作了一种利用介电泳芯片的装置,如图3e所示。在微流控芯片通道内部填充蓝色使用染料,从左侧的入口将细胞样品注入,中间储层用来药物输送,在电极产生的介电泳力作用下对骨髓性白细胞进行捕获。利用介电泳芯片装置对单细胞分析,检测多药耐药(multidrug resistance, MDR)的药物流出功能中单细胞的异质性,并捕获了具有MDR活性的白血病细胞和无MDR活性的白血病细胞,将其与良性白细胞区分。这对未来的医疗试验研究提供了一个确定单细胞水平上MDR抑制的异质性新技术。

综上所述,介电泳技术由于对尺寸相近且节点特性相差较小的微纳尺度物质分离不具有高分辨率,且传统介电泳装置存在高电压条件下易对生物细胞造成损伤,同时有效电场较小,因此可将介电泳技术与微流控技术进行联用,利用微流控技术装置的小型化设计,在低电压条件下产生较高的有效电场,对带有不同尺寸、不同介电特性的混合样品进行精准分离。

### 2.3 电场流动分离技术

场流分离技术最早由Giddings等^[72]^发明,是分析分离领域用来分离大分子胶体和颗粒材料的一种分离方法,随着场流分离技术的不断发展,其也逐渐成为色谱分离体系中一项重要的分离技术。其中电场场流分离技术被越来越多的研究学者使用,其分离原理是通过在分离通道的上下壁(电极)所施加的直流电场或交流电场,对混合带电颗粒进行精准分离^[73]^。该技术可以根据混合样品的大小与带电性质的不同进行分离和聚焦等处理,如图1c所示。传统的直流电场流分离技术是针对通道壁施加固定电压,通道壁表面在直流电场条件下形成双电层,降低通道内有效电场,导致电场场流分离技术的分离效率大大降低。在交流电场流分离技术中,施加的交流电场根据频率调节电场方向,减缓内电极表面形成的双电层,提高通道内有效电场,起到提高分离效率的作用。目前电场场流分离技术可以对颗粒物、生物细胞和外泌体等进行有效分离,被广泛应用于微纳米颗粒和细胞分离等领域。Tasci等^[74]^为了改善纳米颗粒在交流电场场流分离中存在的小尺寸微纳尺度物质的扩散现象,对电参数中的偏置电压进行调节。该团队使用高于50%的偏置电压对50 nm以下颗粒物进行有效分离,结果证明通过调节偏置电压可以减小扩散现象对分离纳米颗粒的影响,提高分离效率。Petersen等^[75]^利用交流电场场流分离技术对外泌体的分离进行研究,以交流电压为常量,流动相为变量的条件下对外泌体进行了分离,证实交流电场流分离技术可以对外泌体进行有效分离。

在电场场流分离技术中,电极容易产生电极极化的现象,限制了电场场流分离技术在微流控芯片中的应用。对此,本课题组^[45]^提出了微颗粒分离“靶式分布”新概念毛细管靶式电场流分离技术,如图4所示。利用离子液体及介孔硅材料界面修饰技术解决了电极极化的问题,实现了在环形通道中对微纳尺度物质的在线分离。通过微流控芯片模拟该体系下颗粒物的运动情况,成功分离不同尺寸的聚苯乙烯颗粒物。该技术解决了传统电场流分离领域中电极极化的问题,并提出了颗粒物的靶式分布和锥形排列新理念,在单细胞分离分析、外泌体分离等具有广阔的应用前景。

**图4:**
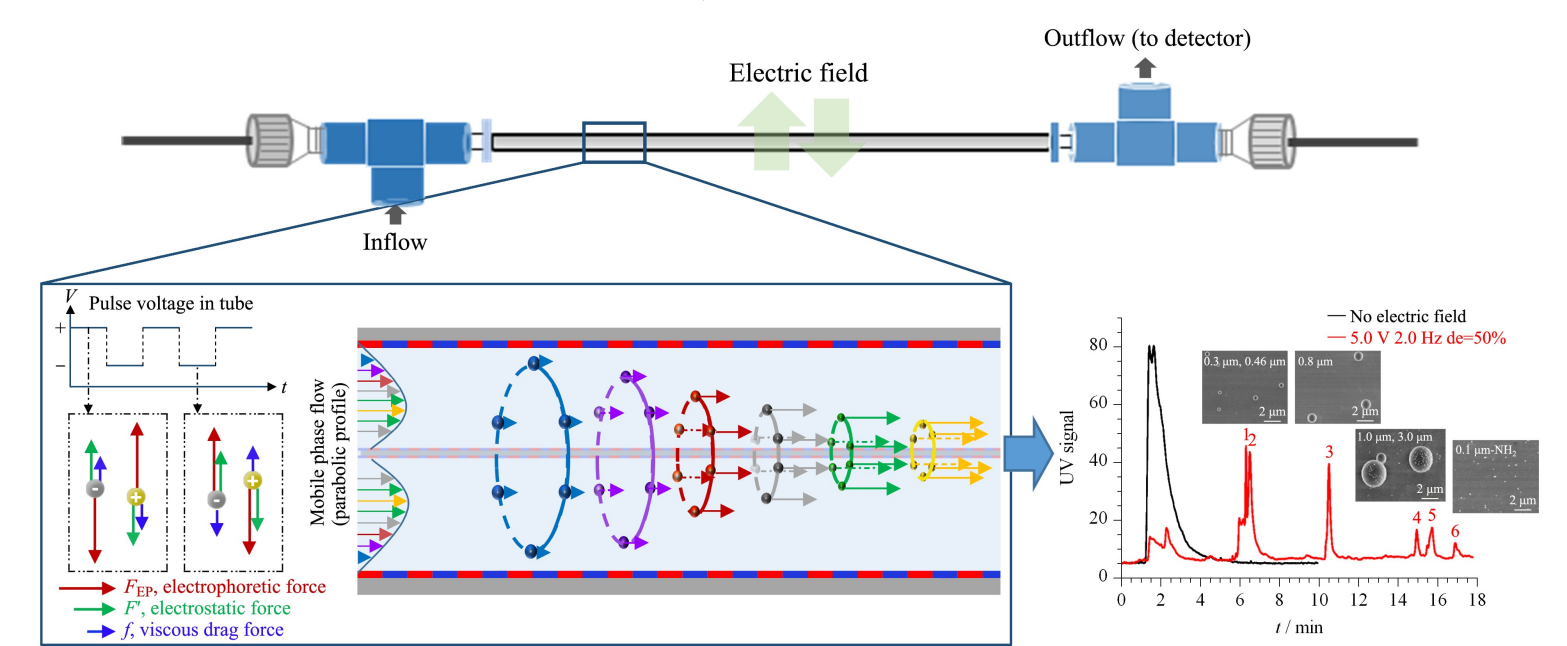
开管式毛细管电场流微分离技术原理示意图^[45]^

### 2.4 电渗分离

感应电荷电渗(induced-charge electroosmosis, ICEO)是导体表面与电场相互作用产生的双电层的扩散层在切向电场作用下产生微旋涡的一种电化学效应。在电场条件下电极表面发生极化现象形成双电层,双电层分为致密层与扩散层,其中扩散层在电场作用下发生移动,致密层中离子不动,进而在电场内形成涡旋,混合样品中不同带电特性的样品将会随着涡旋逐渐向悬浮电极中心移动,根据不同的运动行为,达到分离作用^[64,76,77]^,如图1d所示。基于ICEO的颗粒分离方法具有可调节流型、操作方便、无接触等优点^[78]^。

Chen等^[79]^提出了一种利用诱导电荷电渗透在连续流体中分离颗粒的微流控芯片装置,如图5所示。利用ICEO产生涡旋成功对聚苯乙烯颗粒(polystyrene particle, PS)与二氧化硅微粒颗粒物进行分离,分离效率在99%以上。ICEO对酵母细胞的纯化回收率超过96%。感应电荷电渗透技术在与微流控技术联用后,可以针对不同样品进行聚焦、分离和纯化等实验,为今后生物、医疗和化学领域的微纳尺度物质分离提供一种有效技术支撑。

**图5:**
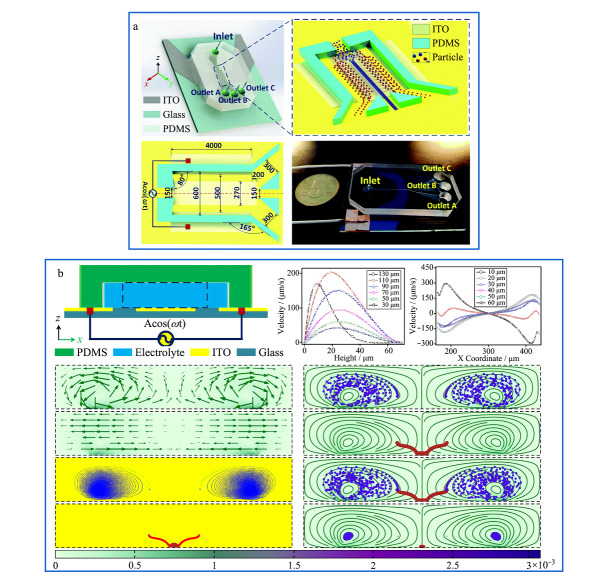
基于诱导电荷电渗透的微流控分离系统

综上描述,电场分离技术的无标记分离、高选择性以及高效分离等特点和微流控装置样品量少、金属污染少、样品制备简单等特性的结合大大提高了对生物细胞和微纳米颗粒的精准分离、捕获及聚焦等效果。在微纳尺度物质分离领域中具有良好的应用前景。

## 3 基于外加磁场的分离技术

近年来,利用外加磁场的作用操纵微颗粒、细胞等微纳米尺度物质分离分析逐渐被重视起来。磁场分离技术中磁性纳米颗粒与目标物可以简单有效的结合,磁场条件下可以对包裹磁性纳米颗粒的样品实现温和、无损和高通量的快速分离^[9,80-82]^,因此在分离微纳尺度物质研究中成为最佳选择之一。同时,在微流控体系下采用磁珠分离方法对微纳尺度物质进行分离也已非常普遍^[83,84]^。越来越多的研究学者将磁场分离技术与微流控技术进行联用,在微流控体系下的磁场分离技术利用磁性颗粒修饰的抗体或官能团,与所需的细胞或蛋白质进行特异性结合,在不同磁场条件下微流控连续分离装置可以分离不同类型的微尺度混合物^[85,86]^,利用磁场与微流控技术的耦合装置可以分离磁性颗粒与非磁性颗粒,以及带有不同性质的磁性颗粒物^[87]^。所以利用磁场分离技术与微流控装置结构进行联用受到国内外研究者的广泛关注。Kumar等^[88]^开发了一种新型的微流控装置,该装置采用永磁体,在0.5~5 mL/h流速范围内研究了11个聚氰胺微粒子对薄微通道壁的无鞘液磁性聚焦。将顺磁性粒子的混合物注入该装置以演示其分选原理。两种不同尺寸的混合磁粒子均沿通道壁排列,在设备前半段达到聚焦,不同尺寸大小目标物位于不同的流线上,在进入膨胀区域时,被分割成明显的流线以达到分离的效果。通过磁场场流分离与微流控装置的联用,达到了高通量(10000 cells/s)和高纯度(98%)的效果。该方法克服了传统磁泳法操作复杂、准备和操作时间长等局限性,将磁场分离技术与微流控技术进行联用,从而对不同尺寸、不同磁性的微纳米颗粒和生物细胞进行分离分析。

Pamme等^[89]^利用特定的微流控装置对磁性纳米颗粒和非磁性纳米颗粒进行连续流动分离,如图6a所示。通过将混合样品注入装置内的分离室,颗粒物在受到垂直向上的磁力作用下,根据颗粒物自身磁化率和大小等性质不同,在不同的层流方向上产生偏转达到分离的效果。利用微流控分离通道设计性强的优点,可以根据混合样品的磁性不同来调节磁铁的具体位置,达到精确分离的效果,分离后的目标物在微流控装置的不同出口处流出,进入到不同的缓冲层,在下游对其进行多步生化处理,为后续实验做准备。

**图6:**
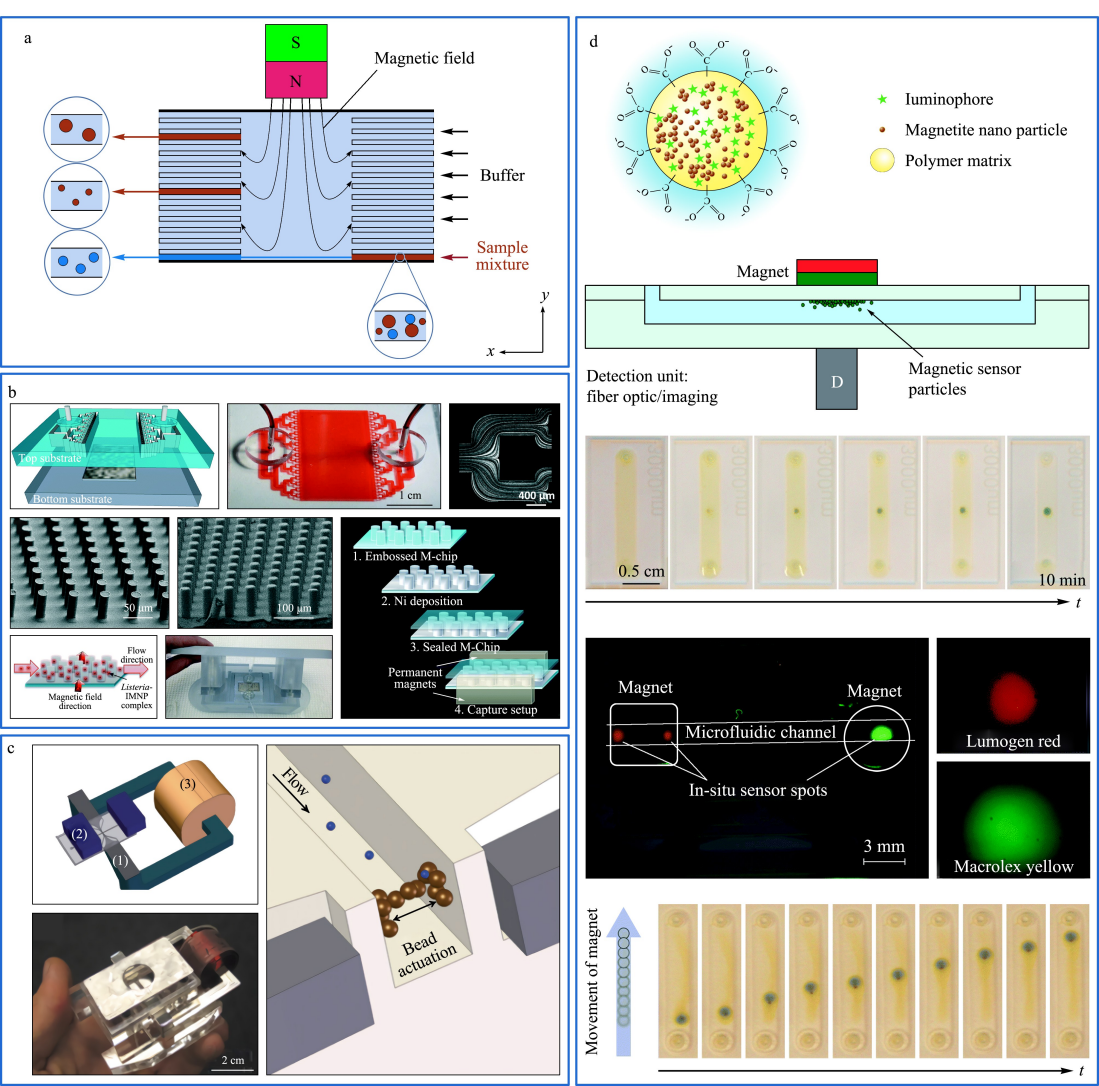
基于磁场的微流控分离系统

Malica等^[90]^以食源性病原体引起的人体感染疾病问题作为出发点,针对单核细胞增生李斯特菌引起的感染对卫生安全构成威胁等问题进行研究,提出了利用磁性纳米粒子(magnetic nanoparticles, MNPs)对其进行免疫磁分离。根据它可以有效对目标细胞进行捕获的优点,与微流控技术耦合制作了一个磁场-微流控芯片装置,如图6b所示,该装置的磁捕获区由多个涂覆了软铁磁镍的圆柱组成,可以产生强大可切换的三维磁陷阱,并利用数值和相关理论分析预测磁陷阱周围的磁场分布,对磁标记的细菌进行高效磁捕获和释放,具有灵活、可定制、低成本等特点,还可以针对各种微米以及亚微米级别目标物分离捕获,对MNPs的最大回收率为91%,活菌的最大捕获效率为30%。该微流控装置可以对食品安全细菌检测提供技术支持。

Moser等^[91]^提出了一种在芯片上捕获蛋白质的磁珠免疫凝集方法,如图6c所示,该方法利用垂直在流动方向上的磁力,使磁性颗粒固定凝集在通道壁上,而磁场梯度的周期性翻转和力场的变化使磁珠在通道内不停地做循环运动,形成更大的磁粒子动态塞,用来捕获流动相中的目标物,并用简单的光学检测方法确定免疫凝集的磁珠量以及浓度,为芯片上蛋白质捕获领域提供了一个更加有效的技术支持。

Ungerbock等^[92]^对磁光传感器粒子(magneto-optic sensor particles, MOSePs)在微流控装置中的实用性进行了评价,如图6d所示。MOSePs可以用于任何带有光学透明微流控芯片的微观氧气成像、多重分析物的并行监测和作为灵活的传感器点监测酶活性,当无法集成传感器层时,也可以在微流控结构中形成固定传感器点。以氧传感器为例,Ungerbock等^[92]^研究了不同直径的MOSePs的积累特性以及在不同流速下原位传感器的稳定性,利用马高列斯荧光黄色染料(MFY)和路玛近红色染料(LR)对磁光传感粒子进行染色,再从外部使用磁铁装置对其进行分离。实验结果证明,MoSePs作为微流控器件中的一部分,促进发光传感器领域的进一步集成。

综上所述,磁场的无标记特性与微流控技术的联用,在微流控芯片装置内部将微纳尺度物质根据自身性质(大小、磁化率)的不同,实现对微纳米颗粒的在线连续分离,达到对目标样品的快速、无损和高效分离和捕获。

## 4 基于外加声场的分离技术

在微纳米尺度下如何实现微颗粒的精准操纵一直是研究的热门。研究发现,施加外场的方式可以实现对微颗粒的操纵,其中运用声场的方式相较于其他外场来说所需能量更小,不会损坏细胞等微纳尺度物质活性和对样品电性和磁性等无特殊要求,因此适用面更为广泛。声场分离技术是指在微流体系统中利用声辐射力(acoustic radiation force, ARF)操纵悬浮液中的微纳尺度物质分离的一种技术^[93]^。ARF的表现形式可以分为体声波(bulk acoustic wave, BAW)或表面声波(surface acoustic wave, SAW)。SAW更容易通过增加频率来调节粒子运动速度,从而操纵粒子的运动,甚至可以驱动流动相。另外,在1988年,Semyonov等^[94]^首次提出声场场流分离技术(acoustic field-flow fractionation, AcFFF)。它可以将液体内部的胶体颗粒、蛋白质和细胞等物质进行聚焦分离处理,利用声辐射力作为驱动流动相中混合颗粒物的外力,根据混合样品中各个微粒的大小、尺寸和密度等性质所产生的运动行为在微流控芯片装置中起到分离作用,以及利用小尺寸颗粒物在流动相中的扩散起到抑制的作用^[95]^,达到精准分离和聚焦的效果,目前广泛应用于细胞筛选、细胞捕获和生物聚合物分离等^[96,97]^。体声波微流控芯片可以利用驻波的声泳力来提高吞吐量,也可以对颗粒物进行聚焦。这两种方法具有细胞损害小、保持细胞完整性和装置便捷的优点。Hwang等^[98]^研制了一种声场场流分离装置,在特定的微流控装置的通道中,沿重力方向上释放超声波驻波并在通道底部形成一个压力节点。同一方向的声场力和重力对小尺寸混合颗粒物起到抑制扩散的作用。通过荧光显微镜的检测观察,该微流控装置成功对1.0、3.5和10 μm的混合荧光颗粒物进行了有效分离。

Jakobsson等^[99]^提出了一种利用超声波驻波在微流控通道内部对红细胞进行聚焦和控制的方法,如图7a所示。红细胞可以用来检测生物细胞的生理状态,但是细胞在流动相中面向激光光源的横截面位置不同,因此相同细胞所处不同的横截面时可能检测出不同的光散射测量值,所以控制红细胞的定向能力对生物医疗分离是有必要的。该团队利用声波对细胞无损害、灵敏性强的特点在微流控芯片中利用驻波声场将红细胞的横截面最小的尺寸方向与声场方向平行。结果表明,有87.8%±3.8%的红细胞可以水平定向,有98.7%±0.3%的红细胞可以垂直定向。该技术对快速发展的流式细胞计数和图像细胞计数都有潜在贡献。

**图7:**
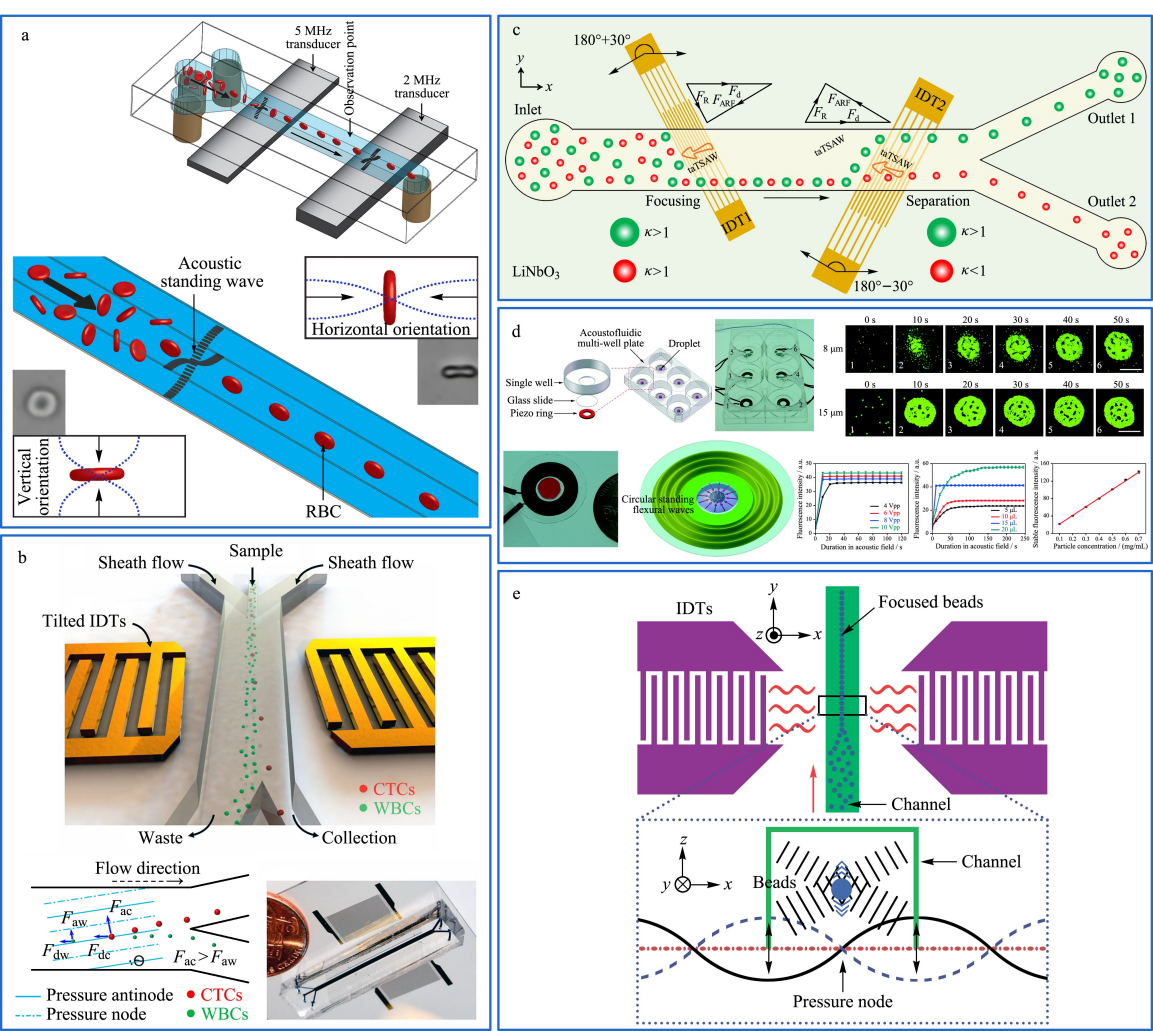
基于声场的微流控分离系统

Li等^[100]^发现从癌症患者的临床样品中筛选循环肿瘤细胞(circulating tumor cell, CTC)存在技术限制、吞吐量不足和缺乏设备长期稳定性等问题,选择利用声场力无标签和无接触分选的优点并结合微流控装置高吞吐量的优势制作了一种倾斜角站立表面声波(standing surface acoustic waves, SSAW)微流控装置,如图7b所示。该装置可以在流速为20 μL/min的条件下将循环肿瘤细胞从白细胞(white blood cells, WBC)中进行有效分离,并且针对癌细胞的回收率可以达到83%~96%,白细胞的去除率达到99%。这种方法适用于分离和白细胞有显著大小和密度差异的癌细胞,利用倾斜角度的驻表面声波(tilted-angle standing surface acoustic waves, taSSAW)装置对临床样本进行高通量分离,从白细胞中分离罕见癌细胞的效率比以往技术具有更高的分离性能,并适用于细胞清洗、细胞同步、血液成分分离和细菌分离等。

Ahmed等^[101]^制作了一种倾斜角度的表面声波(travelling surface acoustic wave, taTSAW)的无鞘液聚焦和连续流中的粒子分离装置,如图7c所示,利用两个互相交叉传感器(interdigitated transducers, IDT)产生与流动方向呈30°的高频倾斜角度表面声波,在不使用鞘液的条件下直接对粒子施加声辐力,分别在194 MHz和136 MHz频率下通过IDTs的激发,粒子被连续聚焦在微通道壁面的一侧,在流速为83.3 mm/s下,taTSAW微流控装置对4.8 μm和3.2 μm颗粒的样品混合物聚焦并分离,分离纯度高达99%。在两个微流控通道出口处颗粒分离率分别为93%和100%。这种方法较其他分离技术相比,不需要额外的鞘层流来进行聚焦处理,在生物和生物医学领域具有潜在的应用价值。

声场也可以对目标物进行聚焦处理,例如Liu等^[102]^利用简单低成本的环形压电传感器制作了一种有压电环阵列组成的声流控多孔板微流控装置,用于在每个板中心快速富集微纳尺度物质,如图7d所示,在玻璃基板上的圆形驻波产生向内的径向声流,诱使微纳尺度物质在声场力的带动下将每个孔中的微纳尺度物质进行富集。实验结果证实,在0.4~30 μm范围内均可进行操纵且具有良好富集效果。具有低成本、低功耗、简单和可控性强等优点,可以在生物和医疗等领域成为强大的工具。

Shi等^[103]^利用驻波表面声波聚焦技术在软光刻法制备的聚二甲基硅氧烷(polydimethylsiloxane, PDMS)通道上进行聚焦实验。如图7e所示,样品在压力驱动力作用下注入微流控通道内,受到两组在悬浮液中相同且延相反方向传播的表面声波形成驻声表面波(SSAWs),通过调节两个IDT释放的波长控制SSAWs的压力节点和反压力节点位置,进而控制其在流体中的周期性压力节点(最小压力)和反正压力节点(最大压力),当通道仅覆盖一个压力节点时,样品受声场影响在中心线处聚焦,由于不同尺寸颗粒物在通道内的运动距离不同,较大尺寸颗粒物比较小尺寸颗粒物有更大的横向位移,该装置对0.87 μm和4.17 μm的乳胶粒子在360 ms内进行了分离。该方法具有操作简单、快速便捷等优势,几乎可用于任何微粒的聚焦。

综上所述,声泳分离技术作为与生物样品非接触的分离方式,在分离生物细胞时具有不破坏生物样品性的优势。将声泳分离技术与微流控技术进行联用可以弥补传统声泳技术装置庞大、操作复杂和对尺寸差异较小的颗粒分辨率低等缺点,从而达到高通量、无损害和操作简单的分离效果,为单细胞分离和捕获等领域提供重要的技术支持。

## 5 总结与展望

利用流动场、电场、磁场和声场等主动场分离技术与微流控技术的联用,可以提高在不同条件下对微纳尺度物质的分离与富集能力。本文综述了4种外加场在微流控技术中的研究进展以及在未来的发展趋势,流场场流分离技术利用温和分离的特性,成功对蛋白质和DNA等物质进行分离,并与其他检测器耦合,对不同样品表征精细化和对生物细胞的分离具有重要意义。在基于电场分离的微流控技术中因物质本身或外部环境影响使其具有带电特性,所以基于电场的微流控技术应用比较广泛,且可实现对微纳尺度物质的无标签、高选择性和高效分离;基于磁场的微流控技术对物质本身磁性要求更为严格,且部分需要对目标物进行标记,难以实现无损分离,因此对分离分析较为脆弱的生物细胞存在限制;将声场和微流控技术联用的装置可以利用声场力对细胞进行精准操控,实现目标物无损伤、高通量、快速的分离。利用主动场分离技术选择性好、分离度高等优点,结合微流控技术的具有微型化、集成化、成本低廉等优势,达到使复杂分析方案合理化,显著减少样品体积和试剂成本,在处理微量样品时具有快速化学分析、高吞吐量和高分辨率的效果,对生物样品的富集浓缩都有较大的研究前景和价值。从应用方面来看,利用微流控芯片实现微纳尺度物质的分离是社会发展的必然趋势,但由于外加场装置普遍都需要复杂庞大的驱动装置,所以实现整个系统的微型化、便捷化仍是迫切的应用需求。随着相关技术的不断发展和进步,未来将会真正实现微型化、集成化的微流控主动场分离技术应用于癌细胞的筛选、癌症的早期检测、微尺度物质的精准分离等各个方面。

## References

[1] Lee A, Elam J W, Darling S B. Environ Sci-Wat Res, 2016, 2(1): 17

[2] El-Sayed I H, Huang X H, El-Sayed M A. Cancer Lett, 2006, 239(1): 129 16198049 10.1016/j.canlet.2005.07.035

[3] Lee W C, Lee B T, Lee S, et al. Microchem J, 2016, 129:219

[4] Jellema L C, Mey T, Koster S, et al. Lab Chip, 2009, 9(13): 1914 19532967 10.1039/b819054b

[5] Di Carlo D. Lab Chip, 2009, 9(21): 3038 19823716 10.1039/b912547g

[6] Helwa I, Cai J W, Drewry M D, et al. PLoS One, 2017, 12(1): 0170628 10.1371/journal.pone.0170628 PMC5256994 28114422

[7] Lee A, Park J, Lim M, et al. Anal Chem, 2014, 86(22): 11349 25317565 10.1021/ac5035049

[8] Fabbri F, Carloni S, Zoli W, et al. Cancer letters, 2013, 335(1): 225 23419522 10.1016/j.canlet.2013.02.015

[9] Veiseh O, Gunn J W, Zhang M. Adv Drug Deliver Rev, 2010, 62(3): 284 10.1016/j.addr.2009.11.002 PMC2827645 19909778

[10] Wang J, Shen Q, Zhang W H, et al. Lwt, 2018, 93:362

[11] Townsend R J, Hill M, Harris N R, et al. Ultrasonics, 2006, 44(8): 467 10.1016/j.ultras.2006.05.025 16782151

[12] He M, Crow J, Roth M, et al. Lab Chip, 2014, 14(19): 3773 25099143 10.1039/c4lc00662c PMC4161194

[13] Petersen K E, Shiri F, White T, et al. Anal Chem, 2018, 90(21): 12783 30346136 10.1021/acs.analchem.8b03146

[14] Luo J H, Muratore K A, Arriaga E A, et al. Anal Chem, 2016, 88(11): 5920 27149097 10.1021/acs.analchem.6b00837 PMC5316477

[15] Bao J M, Wang D D, Li Y X, et al. Chinese Journal of Chromatography, 2017, 35(1): 129

[16] Manz A, Graber N, Widmer H M. Sensor Actuat B-Chem, 1990, 1(1): 244

[17] Cho H, Kim J, Song H, et al. Analyst, 2018, 143:2936 29796523 10.1039/c7an01979c

[18] Shi Y, Shao X G. Chinese Journal of Chromatography, 2019, 37(9): 925 31642295 10.3724/SP.J.1123.2019.01036

[19] Yuan Y X, Fan C, Pan J Z, et al. Chinese Journal of Chromatography, 2020, 38(2): 183 34213167 10.3724/SP.J.1123.2019.05006

[20] Li X, Chen W, Liu G, et al. Lab Chip, 2014, 14(14): 2565 24895109 10.1039/c4lc00350k PMC4106416

[21] Hoshino K, Huang Y Y, Lane N, et al. Lab Chip, 2011, 11(20): 3449 21863182 10.1039/c1lc20270g PMC3379551

[22] Jing T, Lai Z X, Wu L D, et al. Anal Chem, 2016, 88(23): 11750 27797505 10.1021/acs.analchem.6b03370

[23] Lee M G, Shin J H, Bae C Y, et al. Anal Chem, 2013, 85(13): 6213 23724953 10.1021/ac4006149

[24] Valencia P M, Farokhzad O C, Karnik R, et al. Nat Nanotechnol, 2012, 7(10): 623 23042546 10.1038/nnano.2012.168 PMC3654404

[25] Wang Z, Wu H J, Fine D, et al. Lab Chip, 2013, 13(15): 2879 23743667 10.1039/c3lc41343h PMC3740541

[26] Brouzes E, Medkova M, Savenelli N, et al. P Natl Acad Sci USA, 2009, 106(34): 14195 10.1073/pnas.0903542106 PMC2732882 19617544

[27] Thongboonkerd V, Songtawee N, Sritippayawan S, et al. J Proteome Res, 2007, 6(5): 2011 17429990 10.1021/pr060586+

[28] Liao Z R, Li Y R, Gu L, et al. Chinese Journal of Chromatography, 2019, 37(4): 16 10.3724/SP.J.1123.2018.11045 30977335

[29] Wei Z, Li X, Zhao D, et al. Anal Chem, 2014, 86(20): 10215 25252150 10.1021/ac502294e

[30] Kang C M, Joo S, Bae J H, et al. Anal Chem, 2012, 84(2): 901 22148852 10.1021/ac2016322

[31] Lewpiriyawong N, Kandaswamy K, Yang C, et al. Anal Chem, 2011, 83(24): 9579 22035423 10.1021/ac202137y

[32] Munaz A, Shiddiky M, Nguyen N. Sensor Actuat B-Chem, 2018, 275(1): 459

[33] Bi Y, Pan X, Chen L, et al. J Chromatogr A, 2011, 1218(25): 3908 21592484 10.1016/j.chroma.2011.04.065

[34] Wahlund K G, Giddings J C. Anal Chem, 1987, 59(9): 1332 3605623 10.1021/ac00136a016

[35] Wagner M, Holzschuh S, Traeger A, et al. Anal Chem, 2014. 86(11): 5201 24802650 10.1021/ac501664t

[36] Dou H, Li Y, Choi J, et al. J Chromatogr A, 2016, 1465:165 27582461 10.1016/j.chroma.2016.08.062

[37] Kang D, Oh S, Reschiglian P, et al. Analyst, 2008, 133(4): 505 18365121 10.1039/b716851a

[38] Wahlund K G, Zattoni A. Anal Chem, 2002, 74(21): 5621 12433097 10.1021/ac020315s

[39] Osorio-Macías D E, Song D, Thuvander J, et al. J Agric Food Chem, 2020, 68(49): 14564 33236630 10.1021/acs.jafc.9b07251 PMC7735732

[40] Dou H Y, Li Y Q, Choi J, et al. Food Chem, 2016, 192(1): 228 26304341 10.1016/j.foodchem.2015.07.019

[41] Guo P P, Li Y Q, An J X, et al. Carbohyd Polym, 2019, 226(15): 115330 10.1016/j.carbpol.2019.115330 31582064

[42] Ashby J, Schachermeyer S, Pan S, et al. Anal Chem, 2013, 85(15): 7494 23859073 10.1021/ac401485j PMC3815437

[43] Adkins G B, Sun E, Coreas R, et al. Anal Chem, 2020, 92(10): 7071 32316720 10.1021/acs.analchem.0c00406 PMC7340584

[44] Wu R, Zhu K, Zhang X, et al. Anal Chem, 2017, 89(23): 12951 29099175 10.1021/acs.analchem.7b03811

[45] Liu L, Yang C, Liu C, et al. Lab Chip, 2020(20): 3535 32852497 10.1039/d0lc00620c

[46] Zhang W, Wang J, Guo P, et al. Food Chem, 2019, 277(2): 674 30502202 10.1016/j.foodchem.2018.11.033

[47] Zhang X, Li Y, Shen S, et al. TrAC-Trends Anal Chem Chem, 2018, 108:231

[48] Boettcher M, Schmidt S, Latz A, et al. J Phys-Condens Mat, 2011, 23(32): 324101 10.1088/0953-8984/23/32/324101 21795763

[49] Scampicchio M, Wang J, Mannino S, et al. J Chromatogr A, 2004, 1049(1): 189 15499932

[50] Liu Q S, Shi Y Y, Yang W J, et al. J Chromatogr A, 2015, 1399(19): 25 10.1016/j.chroma.2015.04.037 25952665

[51] Ha J W, Hahn J H. Anal Chem, 2016, 88(9): 4629 27056036 10.1021/acs.analchem.6b00789

[52] Gordon M J, Huang X H, Pentoney S L, et al. Electrophoresis, 1988, 242(4876): 224 10.1126/science.242.4876.224 17787651

[53] Ban E, Yoo Y S, Song E J. Talanta, 2015, 141(15): 15 25966374 10.1016/j.talanta.2015.03.020

[54] Nehmé H, Nehmé R, Lafite P, et al. Anal Bioanal Chem, 2013, 405(28): 9159 24057022 10.1007/s00216-013-7332-0

[55] Xu X C, Liu H. Chinese Journal of Chromatography, 2020, 38(10): 47 10.3724/SP.J.1123.2020.03012 34213112

[56] Koenka I J, Sáiz J, Rempel P, et al. Anal Chem, 2016, 88(7): 3761 26926522 10.1021/acs.analchem.5b04666

[57] Thang L Y, See H H, Quirino J P. Anal Chem, 2016, 88(20): 9915 27669824 10.1021/acs.analchem.6b02790

[58] Zhang Y, Zhu L, Zhang Y, et al. J Chromatogr A, 2018, 1555(22): 100 10.1016/j.chroma.2018.04.058 29724645

[59] Jeon H, Kim Y, Lim G. Sci Rep-UK, 2016, 6:19911 10.1038/srep19911 PMC4730231 26819221

[60] Cai Q D, Rong Y Q, Su Y Y, et al. Jouranl of Pharmaceutical Analysis, 2019, 39(6): 975

[61] Pohl H A, Crane J S. J Theor Biol, 1972, 37(1): 1 4652418 10.1016/0022-5193(72)90112-9

[62] Zhou T, Deng Y, Zhao H, et al. J Fluid Eng-T Asme, 2018, 140(9): 1302

[63] Voldman J. Annu Rev Biomed Eng, 2006, 8(1): 425 16834563 10.1146/annurev.bioeng.8.061505.095739

[64] Sasaki N, Kitamori T, Kim H B. Lab Chip, 2006, 6(4): 550 16572218 10.1039/b515852d

[65] Tada S, Nakanishi A, Eguchi M, et al. Biomicrofluidics, 2016, 10(3): 034110 27279934 10.1063/1.4950999 PMC4874929

[66] Zhao K, Li D. Sensor Actuat B-Chem, 2017, 250:274

[67] Zhao K, Larasati L, Duncker B P, et al. Anal Chem, 2019, 91(9): 6304 30977369 10.1021/acs.analchem.9b01104

[68] Kim U, Qian J, Kenrick S A, et al. Anal Chem, 2008, 80(22): 8656 18939853 10.1021/ac8015938 PMC2726846

[69] Khoshmanesh K, Akagi J, Nahavandi S, et al. Anal Chem, 2011, 83(8): 3217 21443166 10.1021/ac2002142

[70] Sun M, Agarwal P, Zhao S, et al. Anal Chem, 2016, 88(16): 8264 27409352 10.1021/acs.analchem.6b02104 PMC5497574

[71] Khamenehfar A, Gandhi M K, Chen Y, et al. Anal Chem, 2016, 88(11): 5680 27149245 10.1021/acs.analchem.5b04446

[72] Ratanathanawongs S K, Shiundu P M, Giddings J C. Colloid Surface A, 1995, 105(2/3): 243

[73] Roda B, Zattoni A, Reschiglian P, et al. Anal Chim Acta, 2009, 635(2): 132 19216870 10.1016/j.aca.2009.01.015

[74] Tasci T O, Johnson W P, Fernandez D P, et al. Anal Chem, 2013, 85(23): 11225 24180262 10.1021/ac401331z

[75] Petersen K E, Manangon E, Hood J L, et al. Anal Bioanal Chem, 2014 10.1007/s00216-014-8040-0 PMC4492512 25084738

[76] Felten M, Staroske W, Jaeger M S, et al. Electrophoresis, 2010, 29(14): 2987 10.1002/elps.200700844 18655037

[77] Chen X, Ren Y, Liu W, et al. Anal Chem, 2017, 89(17): 9583 28783330 10.1021/acs.analchem.7b02892

[78] Ren Y, Liu J, Liu W, et al. Lab Chip, 2016, 16:2803 27354159 10.1039/c6lc00485g

[79] ChenX M, Ren Y K, Hou L K, et al. Nanoscale, 2019, 11(13): 6410 30888357 10.1039/c8nr09148j

[80] Ngamsom B, Esfahani M M N, Phurimask C, et al. Anal Chim Acta, 2016, 918(28): 69 27046212 10.1016/j.aca.2016.03.014

[81] Jia Z, Liang Y, Xu X, et al. Cell Biol Int, 2018, 41(3): 657 10.1002/cbin.10903 29068101

[82] Müller P, Gaebel R, Lemcke H, et al. Biomaterials, 2017, 135:74 28494265 10.1016/j.biomaterials.2017.05.002

[83] Mizuno M, Yamada M, Mitamura R, et al. Anal Chem, 2013, 85(16): 7666 23875607 10.1021/ac303336f

[84] Latham A, Freitas R, Schiffer P, et al. Anal Chem, 2005, 77(15): 5055 16053322 10.1021/ac050611f

[85] Iiguni Y, Tanaka A, Kitagawa S, et al. Anal Sci, 2016, 32(1): 41 26753704 10.2116/analsci.32.41

[86] Wu J, Yan Q, Xuan S, et al. Microfluid Nanofluid, 2017, 21(3): 47

[87] Leshuk T, Holmes A, Ranatunga D, et al. Environ Sci-Nano, 2018, 5(2): 509

[88] Kumar V, Rezai P. Biomedical Microdevices, 2017, 19(2): 39 28466285 10.1007/s10544-017-0178-z

[89] Pamme N, Manz A. Anal Chem, 2004, 76(24): 7250 15595866 10.1021/ac049183o

[90] Malica L, Zhang X, Brassard D, et al. Lab Chip, 2015, (15): 3994 26346021 10.1039/c5lc00852b

[91] Moser Y, Lehnert T, Gijs M A M. Lab Chip, 2009, 9(22): 3261 19865734 10.1039/b907724c

[92] Ungerbock B, Fellinger S, Sulzer P, et al. Analyst, 2014, 139(10): 2551 24695649 10.1039/c4an00169a

[93] Glynne-Jones P, Boltryk R J, Harris N R, et al. Ultrasonics, 2010, 50(1): 68 19709711 10.1016/j.ultras.2009.07.010

[94] Semyonov S N, Maslow K I. J Chromatogr A, 1988, 446(17): 251

[95] Townsend R J, Hill M, Harris N R, et al. Ultrasonics, 2008, 48(6/7): 515 18664397 10.1016/j.ultras.2008.06.005

[96] Johansson L, Enlund J, Johansson S, et al. J Micromech Microeng, 2012, 22(2): 025018

[97] Hawkes J J, Barber R W, Emerson D R, et al. Lab Chip, 2004, 4(5): 446 15472728 10.1039/b408045a

[98] Hwang J Y, Youn S, Yang I H. Anal Chim Acta, 2018, 1047(24): 238 30567656 10.1016/j.aca.2018.09.056

[99] Jakobsson O, Antfolk M, Laurell T. Anal Chem, 2014, 86(12): 6111 24863098 10.1021/ac5012602

[100] Li P, Mao Z, Peng Z, et al. P Natl A Sci India B, 2015, 112(16): 4484

[101] Ahmed H, Destgeer G, Park J, et al. Anal Chem, 2018, 90(14): 8546 29911381 10.1021/acs.analchem.8b01593

[102] Liu P, Tian Z H, Hao N J, et al. Lab Chip, 2020, 20(18): 3399 32779677 10.1039/d0lc00378f PMC7494569

[103] Shi J, Mao X, Ahmed D, et al. Lab Chip, 2008, 8(2): 221 18231658 10.1039/b716321e

